# Supplementary material for: Involvement of Organic Anion Transporters in the Pharmacokinetics and Drug Interaction of Rosmarinic Acid
Source: Pharmaceutics. 2021 Jan 9;13(1):83. doi: 10.3390/pharmaceutics13010083 (PMC7828042; doi:10.3390/pharmaceutics13010083)
Supplement: Supplementary file 1 [file pharmaceutics-13-00083-s001.pdf]

pharmaceutics

MDPI

Supplementary Materials: Involvement of Organic Anion Transporters in the Pharmacokinetics and Drug Interaction of Rosmarinic Acid

Yun Ju Kang[1], Chul Haeng Lee[2], Soo-Jin Park[3], Hye Suk Lee[4], Min-Koo Choi[2,*], Im-Sook Song[1,*]

1. LC-MS/MS Analysis of Rosmarinic Acid, Probenecid, Furosemide, and Valsartan

1.1. Precision and Accuracy

The inter-day and intra-day precision and accuracy were assessed using three different concentrations of quality control (QC) samples of rosmarinic acid, probenecid, furosemide, and valsartan, respectively (Table S1). The results showed that the inter-day and intra-day accuracy for rosmarinic acid, probenecid, furosemide, and valsartan was ranged from 90.9% to 112.7%, and the inter-day and intra-day precision (expressed as coefficient of variance (CV, %)) for rosmarinic acid, probenecid, furosemide, and valsartan was below 12.5% (Table S1).

Table S1. Intra- and inter-day precision and accuracy of rosmarinic acid, probenecid, furosemide, and valsartan.

Analytes	QC Concentration (μM)	Intra-Day (*n* = 6)			Inter-Day (*n* = 5)		
		Accuracy (%)	SD	Precision (CV, %)	Accuracy (%)	SD	Precision (CV, %)
Rosmarinic acid	0.1	96.3	12.1	12.5	90.9	6.8	7.5
	15	93.9	7.6	8.1	90.8	8.1	8.9
	300	112.6	3.6	3.2	111.9	5.3	4.8
Probenecid	0.15	101.8	6.7	6.6	104.7	7.8	7.4
	25	107.3	7.6	7.1	112.5	4.3	3.8
	500	102.9	7.2	7.0	109.7	2.6	2.4
Furosemide	0.03	111.8	12.1	10.8	98.3	7.5	8.4
	0.3	101.9	5.4	5.3	92.3	2.9	3.2
	3	110.8	3.3	3.0	109.4	5.3	4.9
Valsartan	0.15	112.7	3.8	3.3	111.7	5.8	5.2
	1.5	109.2	6.4	5.9	112.2	2.9	2.6
	15	104.9	7.9	7.5	110.6	3.2	2.9

QC, quality control; SD, standard deviation; CV, coefficient of variance.

1.2. Matrix Effect and Extraction Recovery

Matrix effects were monitored by dividing the peak areas of QC samples in blank plasma by corresponding QC samples in neat solution and extraction recovery was calculated by comparing the peak areas of QC samples following sample preparation procedure with those of the pre-extraction spiked samples.

As results, the matrix effects of rosmarinic acid, probenecid, furosemide, and valsartan in the three different concentrations of QC samples prepared by protein precipitation methods ranged from 71.8 to 90.1% with CVs of below 11.4% (Table S2). The extraction recoveries of rosmarinic acid, probenecid, furosemide, and valsartan in the three different concentrations of QC samples ranged from 85.1% to 93.71% with CVs of below 7.2% (Table

S2). These results indicate that no significant interference occurred during the ionization and protein precipitation methods.

Table S2. Matrix effects and extraction recoveries of rosmarinic acid, probenecid, furosemide, and valsartan.

Analyte	QC Concentration (μM)	Matrix Effect (%) Mean ± SD (*n* = 4)	CV (%)	Extraction Recovery (%) Mean ± SD (*n* = 4)	CV (%)
Rosmarinic acid	0.1	84.0 ± 7.3	8.6	89.8 ± 5.7	7.1
	15	85.1 ± 2.0	2.3	90.2 ± 6.5	7.2
	300	71.8 ± 2.4	3.4	85.3 ± 1.9	2.3
Probenecid	0.15	79.7 ± 5.7	7.1	85.1 ± 3.2	3.8
	25	81.0 ± 3.4	4.2	91.2 ± 4.5	5.0
	500	82.0 ± 2.9	3.5	93.7 ± 3.9	4.1
Furosemide	0.03	83.5 ± 9.5	11.4	86.7 ± 5.6	6.5
	0.3	86.1 ± 9.1	10.6	89.3 ± 3.7	4.1
	3	90.1 ± 6.4	7.1	87.9 ± 4.1	4.7
Valsartan	0.15	82.1 ± 1.3	1.5	85.4 ± 3.2	3.7
	1.5	77.5 ± 1.1	1.4	90.1 ± 1.4	1.6
	15	77.5 ± 0.3	0.4	87.3 ± 0.5	0.6

QC, quality control; SD, standard deviation; CV, coefficient of variance

1.3. Stability

The stability of rosmarinic acid, probenecid, furosemide, and valsartan in the rat plasma was tested from QC samples exposed to three different conditions. Short-term stability was calculated by comparing QC samples that were stored for 4 h at 25 °C before sample preparation with the untreated QC samples in rat blank plasma. Post treatment stability was evaluated by comparing the post-preparative QC samples maintained in the autosampler at 6 °C for 24 h with the untreated QC samples. The freeze–thaw cycle stability was analyzed by comparing QC samples that underwent three freeze–thaw cycles (-80 °C to 25 °C and standing for 3 h at 25 °C defined as one cycle) with the untreated QC samples.

Rosmarinic acid, probenecid, furosemide, and valsartan in plasma samples were stable for up to 4 h at 25 °C with RSDs of below 8.9% and for 24 h post treatment stability measurement at 6 °C with RSDs of below 9.2% (Table S3). No significant degradation occurred in the three different concentrations of QC samples of rosmarinic acid, probenecid, furosemide, and valsartan from the three freeze-thaw cycle stability (Table S3), suggesting that rosmarinic acid, probenecid, furosemide, and valsartan had no stability issues during analysis.

Table S3. Short-term stability, post-treatment stability, and freeze-thaw cycle stability of rosmarinic acid, probenecid, furosemide, and valsartan.

Analyte	QC Concentration (μM)	Short Term Stability (4 h, 25 °C)		Post-Treatment Stability (6 °C, 24 h)		Free-Thaw Cycle Stability (−80 °C /25 °C, 3 Cycles)	
		Mean ± SD (*n* = 3)	CV(%)	Mean ± SD (*n* = 3)	CV(%)	Mean ± SD (*n* = 3)	CV(%)
Rosmarinic acid	0.1	96.1 ± 6.8	7.1	110.8 ± 3.6	3.3	95.3 ± 10.3	11.0
	15	99.6 ± 1.2	1.2	97.7 ± 7.3	7.5	103.4 ± 4.2	4.0
	300	11.5 ± 2.6	2.3	112.3 ± 4.3	3.8	94.2 ± 5.7	6.0
Probenecid	0.15	86.8 ± 6.5	7.5	98.4 ± 3.9	4.0	108.9 ± 1.0	0.9
	25	98.5 ± 2.2	2.3	100.5 ± 2.3	2.3	111.7 ± 1.3	1.2

	500	110.2 ± 4.2	3.8	98.0 ± 2.6	2.6	111.2 ± 0.9	0.8
Furosemide	0.03	90.8 ± 6.5	7.2	89.3 ± 8.2	9.2	97.9 ± 3.1	3.2
	0.3	109.3 ± 9.7	8.9	99.4 ± 8.9	9.0	105.4 ± 7.5	7.1
	3	97.1 ± 3.6	3.7	92.3 ± 4.0	4.3	99.4 ± 7.2	7.2
Valsartan	0.15	98.9 ± 4.3	4.3	110.5 ± 3.7	3.4	109.9 ± 2.6	2.4
	1.5	96.3 ± 2.8	2.9	110.2 ± 1.8	1.6	111.2 ± 3.2	2.9
	15	89.5 ± 3.3	3.7	109.1 ± 2.3	2.1	111.4 ± 1.6	1.6

QC, quality control; SD, standard deviation; CV, coefficient of variance
